# Immunization Against Group B Streptococci vs. Intrapartum Antibiotic Prophylaxis in Peripartum Pregnant Women and their Neonates: A Review

**DOI:** 10.7759/cureus.1775

**Published:** 2017-10-13

**Authors:** Mariya Kristeva, Catherine Tillman, Ashley Goordeen

**Affiliations:** 1 University of Central Florida College of Medicine, Orlando, USA

**Keywords:** group b streptococcus, immunization, antibiotic prophylaxis

## Abstract

Group B streptococcus (GBS) is an organism that has an immense global impact on neonatal morbidity and mortality. GBS is known to colonize the gastrointestinal and genitourinary tracts of infected pregnant women. Transmission to the neonate is achieved during labor and delivery. Complications of neonatal infection include sepsis, meningitis, and pneumonia. Preventative measures are needed to reduce the disease burden. The current method of prophylaxis being utilized is intrapartum antibiotics. Though effective, it presents limitations that include a required, lengthy duration of treatment, increased antibiotic resistance, and lack of late-onset disease coverage. Recent studies are exploring alternative methods of prophylaxis such as vaccination. Vaccination provides coverage of both early and late-onset disease to infected women who are unable to complete the required four hours of antibiotic course intrapartum, women with resistance to antibiotic infections, and women delivering in resource-poor areas without access to antibiotics. An investigational CRM197 conjugated trivalent vaccine has shown a good immunogenicity profile against the three most prevalent serotypes. The vaccine induced antibodies freely cross the placenta and persist in a neonate for up to 90 days after birth. Additionally, the vaccine possesses a safety profile free of any serious systemic adverse effects and only mild pain at the injection site. Future studies should focus on investigating the effect of the vaccine on maternal rectovaginal acquisition and subsequent vaginal colonization, as well as the expansion of vaccine coverage to all 10 serotypes identified worldwide in order to facilitate its use in resource-poor areas of the world with inadequate antibiotic availability. If available, such a vaccine can serve as an effective supplement to current guidelines of antibiotic prophylaxis against GBS in peripartum women and their neonates.

## Introduction and background

Group B streptococcus (GBS), an encapsulated gram-positive diplococcus, is a pathogen known to cause disease in all age groups but can be especially severe in newborns. GBS inhabits the gastrointestinal and genitourinary tracts of about 15-40% of pregnant women [1]. Transmission of GBS to the neonate can occur during labor or via fetal membrane rupture. Presentation at, or within 24 hours of birth to the sixth day of life is known as early-onset disease (EOD). It has an incidence of about 0.25 cases per 1000 live births [2]. Late-onset disease (LOD) ensues at four to five weeks of life and has an incidence of 0.27 cases per 1000 live births. GBS infections are the leading cause of death by infectious disease in the neonate. Meningitis, sepsis, and pneumonia are diseases implicated in GBS infection. Approximately one-half of maternal GBS infections lead to newborn colonization and 2-3% result in either fetal and neonatal mortality, neonatal infection, or loss of pregnancy [1].

Pregnant women are screened for GBS in weeks 35-37 of gestation to determine whether they should be administered prophylactic antibiotics at the time of labor to prevent vertical transmission to the neonate by diminishing vaginal colony count. This intervention results in a significant risk reduction of all infections but has no impact on late-onset GBS infection [3]. In addition to this limitation, though antibiotic prophylaxis has been efficacious, maximum benefit from this intervention is dependent on receiving antibiotics for a period of four hours. Thus, women with a shorter delivery course are unable to obtain the proper course of antibiotics and have an increased incidence of neonatal sepsis [4]. Due to the impact that GBS has on neonatal morbidity and mortality, an alternative means of prevention is necessary.

Vaccination against GBS is a modality being studied to prevent GBS infection in both neonates and pregnant women. The development of a vaccine for GBS offers protection against both early-onset and late-onset infection. The vaccines developed are based on a capsular polysaccharide produced from human GBS isolates. These vaccines are effective, safe, and immunogenic [5]. Another advantage of vaccination is its role in a decrease of the emergent antibiotic resistance seen with the use of prophylactic antibiotics. In those cases, where antibiotic prophylaxis is not suitable, it offers an alternative means of disease prevention in peripartum women and neonates. To gain a better understanding of the effectiveness of immunization of pregnant women against GBS and of adverse events related to the immunization versus intrapartum antibiotic prophylaxis, we searched PubMed for  "Peripartum Period"[MeSH]* AND ("Streptococcal Infections"[MeSH]) OR "Streptococcus agalactiae"[MeSH]) AND ("Immunization"[MeSH] OR "Antibiotic Prophylaxis"[MeSH]) and filtered to 'Reviews'. The search returned 100 articles. After careful examination of the review articles, we identified the three most recent studies of a candidate vaccine. Our objective is to investigate the appropriateness of intrapartum antibiotic prophylaxis to prevent group B streptococcal disease in peripartum women and their neonates and to explore the feasibility of supplementing antibiotic prophylaxis by a safe and effective vaccine.

* [MeSH] = medical subject headings

## Review

Current guidelines

Current Centers for Disease Control and Prevention (CDC) guidelines for GBS screening in pregnant women indicate vaginal-rectal testing for all pregnant women at 35 to 37 weeks’ gestation [6]. Following positive results, women should be treated intrapartum for at least four hours with penicillin, first line, or cefazolin, erythromycin, or clindamycin for allergic cases. The effectiveness of current GBS treatment in the prevention of neonatal sepsis is dependent on the ability of current testing to identify women currently carrying GBS and the success of current treatment in eradicating the infection. Additionally, other factors can complicate appropriate treatment including preterm labor, precipitous labor, failure to seek prenatal care, and medication allergies.

Polymerase chain reaction (PCR) analysis or culture are acceptable screening methods on both vaginal and perianal specimens, per CDC recommendations. A multicenter evaluation looked at the diagnostic accuracy of PCR analysis via Xpert GBS®(Cepheid, Sunnyvale, CA) [7]. Using an experimental design, the study compared an assay for group B streptococcus, the Xpert GBS LB assay to the gold standard of broth-enriched culture. The study utilized three trial sites and collected 826 samples from pregnant women between 35 and 37 weeks gestation. The study was ultimately double-blinded, as neither the patient nor the experimenter was aware of the GBS status, and the PCR analysis was run before the culture was completed. No outside funds were utilized for this experiment.  It was determined that PCR testing using the Xpert GBS assay has a sensitivity of 99.0%, specificity of 92.4%, positive predictive value (PPV) of 80.0%, and negative predictive value (NPV) of 99.6%. The high negative predictive values are important for adequately ruling out patients, without leaving GBS positive mothers with false negatives. This study appropriately reflects test results due to a 95% confidence interval (CI), large sample size, and use of an appropriate gold standard. Despite increased cost, the PCR test has an increased turnaround time. Women can be tested closer to delivery, receiving results and treatment sooner. The faster turnaround benefits the mothers who are negative for GBS at 35 weeks but may become infected later in the pregnancy, or those who are receiving testing later in the pregnancy. While the results may be less applicable to patients receiving testing appropriately (three to five weeks prior to delivery), the faster molecular detection method can be applied when the GBS status is unknown and closer to expected delivery or in preterm delivery. The study has high external validity and can practically be applied. Overall, the study concluded that molecular testing is highly sensitive, appropriate, and can reduce the rate of early-onset GBS infections through faster access to results.

Wollheim, et al., conducted a similar study, setting out to explore the effectiveness of PCR assay in comparison to the gold standard culture. They utilized an experimental approach, comparing culture with supplemented gentamicin, nalidixic acid, and sheep blood agar to PCR assay [8]. Double blinding was utilized as the patient and experimenter were blinded to the GBS status of each patient. This study looked at 204 pregnant women visiting the obstetrics and gynecology unit during 35 and 37 weeks gestation, no other inclusion or exclusion criteria were utilized. Samples were collected vaginally and peri-anally. The population was appropriate and equal, including a variety of ages, sociodemographic information, parity, and prevalence of abortions. The gold standard comparison is appropriate via GBS culture. The sensitivity was found to be 100%, the specificity 95.6%, the PPV 86.8%, and the NPV 100%. The values from this study closely support the previous and the authors concluded that PCR is fast and accurate in detecting GBS positive mothers. Limitations include a smaller sample size taken from one hospital site. PCR testing looks at deoxyribonucleic acid (DNA) and non-viable bacteria colonies, leading to a lower positive predictive value (PPV) than a negative predictive value (NPV). The applicability of this test to patients is going to be site dependent based on a cost analysis, as PCR methods have an initially higher cost requirement.

PCR is a suitable substitution for GBS testing over gold standard culture methods. The ideal test is a highly sensitive test with a high negative predictive value to include as many GBS positive women as possible. While the treatment is inexpensive and low risk, the effects of not treating a GBS positive women can be very dangerous for the neonate. As PCR is associated with a higher cost, routine use of the test will be on a hospital by hospital basis, but important for women with an unknown GBS status near the time of delivery.

The success of testing is dependent on appropriate usage in the population. Currently, 90% of women are screened for GBS with 62% screened between 35-37 weeks gestation (the others being tested before the 35-week recommendation). Results were available at delivery in 98% of cases [9].  While screening is provided in a high percentage of the patients, early testing is concerning for late GBS colonization.

Additionally, the effectiveness of current antibiotic recommendations at eliminating the GBS infection influences the overall efficacy of current guidelines. CDC guidelines indicate best practice for treatment is penicillin given intrapartum for at least four hours [6]. Scasso, et al., conducted a prospective study, collecting data from 60 women over the course of one year at Pereira Rossell Hospital in Uruguay to evaluate the effectiveness of penicillin intrapartum antibiotic prophylaxis (IAP) treatment [4]. Inclusion criteria included active labor, singleton pregnancy at 37 weeks or greater, and were GBS positive between 35 and 37 weeks. Exclusion criteria included precipitous delivery, current antibiotic treatment, penicillin allergy, and renal disease. The study demonstrates that penicillin is effective when given as intrapartum antibiotic prophylaxis (IAP) for four hours, with 88% of patients completely resolving the GBS infection. Appropriate treatment fails to treat 12% of patients that are GBS positive. Giving penicillin IAP for less than the four hours dramatically decreases the effectiveness against GBS. After two hours of IAP, only 53% of patients were GBS negative. The risk of infection with GBS is reduced by 75% with an adequate four hours of IAP treatment compared to only two hours of IAP. The research concluded that four hours of IAP, minimum, is required to appropriately treat GBS using penicillin. Additionally, the study demonstrated that in precipitous labor, it can still be useful to provide IAP, as 53% of women were successfully treated with two hours of treatment.

Limitations of this study include solely looking at the effectiveness of penicillin. The population of women with a penicillin allergy is left out of consideration in this article, and increasing resistance of GBS to antibiotics used in place of penicillin is rising. There was some selection bias in excluding women in precipitous labor. Expanding the data to preterm women and women in precipitous labor would provide a more accurate picture, applicable to pregnant women outside of the standard conditions.

While guidelines recommend delivering after intrapartum antibiotic prophylaxis (IAP) for at least four hours, in practice, only about 50% of patients receive the full four hours of treatment. When considering the combination of appropriate drug choice, route of administration, dosage, and timing, only 36% of pregnant women received the correct treatment [9]. While penicillin is an effective treatment IAP for four hours, the lack of consistent and appropriate use demands stricter regulations or is a potential opportunity for vaccines to take out some of the human error.

Many patients have an allergy to penicillin, indicating the importance of, alternate antibiotic selections. For patients without a severe penicillin allergy, it is recommended to provide cefazolin [6]. For those with severe penicillin allergies, clindamycin, erythromycin, and vancomycin can be utilized. A seven-year surveillance study looked at GBS resistance as well as contributions to EOD and LOD in the neonate [10]. Ten hospitals were utilized in Italy to collect a questionnaire from 3,501 patients during the years 2008 to 2009, no other exclusion criteria were mentioned. There were 99 neonatal GBS infections during 2007 and 2010. The questionnaire looked at maternal data, including colonization, gestation at delivery, method of delivery, and IAP, as well as neonatal data, including onset of symptoms, clinical manifestations, and site of GBS isolation. There was no blinding of the data. The results showed screening neonates in 96% of the cases and IAP was delivered appropriately in 37.5% of the cases. Thirty percent of neonatal GBS infections were in preterm babies. Of full-term deliveries, which resulted in EOD, 81% of the mothers were tested for GBS, 46% tested negative. Seventy-six percent of the patients that tested positive, did not receive the complete IAP treatment. LOD was most commonly caused by GBS internationally diffused clonal complex 17. This study also found erythromycin and clindamycin resistance rates to be 17% and 15%, respectively.

The University Hospital of Geneva also showed that there is a high resistance of GBS to clindamycin and erythromycin at 28% and 30% resistance [11-12]. The rate of co-resistance was 92%, indicating the alternative cannot be utilized in the majority of resistant cases. The resistance of GBS to vancomycin was not detailed in this study, another alternative to treatment, although less commonly used. The research concludes even perfect adherence to CDC guidelines would not prevent all neonatal GBS disease and vaccination may be a possible solution. Despite this, correcting lack of adherence to guidelines would further reduce the percentage of EOD in neonates. Resistance rates in the United States may vary from Italy. There is a small sample size of GBS neonatal infections to evaluate. There could also be an element of response bias with the return of the questionnaire. Women who felt their care was inadequate may be more likely to respond. There is external validity as the sample used is representative of the population, including preterm, precipitous labor, and term delivery, both vaginally and through caesarian.

Overall, current guidelines are very effective at identifying GBS positive women and treating the infection when performed correctly, using penicillin IAP for four hours of treatment. The main concern with current management lies in cases of preterm delivery, precipitous delivery, and women with penicillin allergies. In these cases, vaccines may better address the GBS infection, providing prophylactic treatment of neonatal infection and potential sepsis.

Vaccine

There are ten serotypes of GBS identified [13]. Historically, the GBS isolated from infants less than seven days old and peripartum pregnant women were serotypes Ia, Ib, II, and III [7]. However, the most prevalent serotypes in 1980 were Ia, III, and V [14-15]. It is important to take these shifts in serotype predominance into considerations when designing a vaccine against GBS infection as high titer antiserum is serotype specific and shows no cross-reactivity with heterologous antisera.

We reviewed a randomized controlled trial published in 2016 by Donders, et al. The study was a phase two, randomized, observer-blind, multicenter study conducted in Belgium and Canada between September 2011 and October 2013 and was supported by Novartis Vaccines and Diagnostics. 60 healthy pregnant women, 18–40 years old at 24–35 weeks of gestation with no history of hypersensitivity to previous vaccines, alteration of immune function, high-risk pregnancy or receipt of any other investigational agent or intervention anticipated during the study participated in the study. The primary objective of this study was to evaluate the placental transfer of GBS-specific antibodies to neonates born to women administered an investigational trivalent CRM197-conjugated group B streptococcal vaccine or a placebo. The secondary objective was to evaluate the kinetics of GBS serotype-specific antibodies three months after delivery and antibody concentrations in women 30 days post vaccination. As an additional objective, the effect of time from vaccination to delivery on maternal and infant antibody concentrations at delivery and birth was assessed. Women were allocated to receive either the group B streptococcal vaccination or placebo injection. Blood samples were taken on day one and day 30 post vaccination, at delivery, and at 13 weeks postpartum. Blood samples were taken from cord blood at delivery and at three months of age to analyze neonatal immunogenicity objectives. Group B streptococci–specific antibody ratios between mother and infant were in the range of 0.68– 0.81 across the three serotypes for the neonates born to women who received the vaccine (serotype Ia: 0.81 [95% CI 0.72–0.91], serotype Ib: 0.77 [95% CI 0.62–0.97], and serotype III: 0.68 [95% CI 0.59–0.75]). In infants, the persistence of maternally transferred GBS-specific antibody was measured three months after birth. Antibody concentrations decreased after birth and by day 91 were 22–25% of the levels measured at birth. Administration of the investigational trivalent group B streptococcal vaccine to pregnant women had no side effects in the women or their infants and antibodies persisted for at least 91 days in infants of vaccinated women [13].

The same vaccine was investigated by Madhi, et al., who conducted a phase 1b/2, randomized, observer-blind placebo-controlled single-center study of an investigational trivalent GBS vaccine in healthy non-pregnant women and a dose-ranging study in healthy pregnant women [16]. This study was supported by Novartis Vaccines and Diagnostics division and conducted at the Chris Hani Baragwanath Academic Hospital in Soweto, South Africa. Participants were 320 healthy non-pregnant and 60 pregnant at 28–35 weeks gestation, aged between 18 and 40 years. Pregnant women were randomly assigned to receive one injection at 28–35 weeks gestation of 0·5 μg, 2·5 μg, or 5·0 μg of the non-adjuvanted investigational vaccine (for each serotype), or placebo. Reactogenicity was monitored for a week and adverse events and infant safety were recorded throughout the study. The primary outcomes were safety and GBS-specific antibody response after the two injections for nonpregnant women and selection of one vaccine dose based on analysis of serotype-specific antibody responses at delivery (+72 hours) for use in subsequent studies. Among the pregnant women 54–71% experienced one systemic adverse effect of which 4% of the experiences were severe. For nonpregnant women, serotype-specific antibody concentrations were significantly higher than placebo. Pregnant women in all vaccine groups had significantly higher serotype-specific antibody concentrations than the placebo group at delivery (serotype Ia were 11 μg/mL [95% CI 7·0–18] for the GBS vaccine 0·5 μg group, 18 μg/mL [11–29] for the GBS vaccine 2·5 μg group, 22 μg/mL [13–35] for the GBS vaccine 5·0 μg group, and 0·64 μg/mL [0·42–0·98] for the placebo group). Serotype-specific antibody concentrations did not differ significantly between the vaccine doses at any of the measured time points (p>0·05). The vaccine was well tolerated and induced capsular-specific antibody responses in both nonpregnant and pregnant women. Maternal vaccination led to higher GBS serotype-specific antibody concentrations in infants than placebo, with both interventions resulting in similar safety profiles [16].

An American study of an investigational vaccine was conducted by Baker, et al., who examined a type III Group B streptococcal polysaccharide-tetanus toxoid conjugate vaccine [14]. The study was supported by the National Institutes of Health, National Institute of Allergy & Infectious Diseases. The objective of the study was to explore the safety and immunogenicity GBS type III CPS-TT conjugate vaccine in pregnant women. This prospective, randomized, double-blind, placebo-controlled trial enrolled 40 “low risk for obstetrical complications” women between 18 and 45 years of age and 30–32 weeks gestation and their newborns. Women were immunized with a single intramuscular dose of III-TT or saline placebo and instructed to maintain a temperature and symptom diary for a week after the injection to document any adverse effects. Blood was collected before the immunization, four weeks after the immunization, at delivery, and at two months postpartum. Cord blood was collected at birth and infant blood was collected at one and two months of age. Infants were followed until six months of age to monitor for any manifestation of GBS disease. The III-TT conjugate vaccine was well tolerated and no vaccine-associated serious adverse effects occurred. 65% of women reported mild local pain at the injection site and 5% reported moderate pain compared with 40% of placebo recipients. Vaccine recipients had more than 50-fold increases in III capsular polysaccharide-specific (CPS) IgG one month after the immunization, at delivery, and two months postpartum. Placental transport of III CPS-specific IgG allowed for good infant cord values and persistence of serum concentrations at approximately 30% of cord values at two months of age. In summary, GBS III-TT conjugate vaccine was well-tolerated with no serious adverse effects and mild injection site pain. It induced high III CPS-specific IgG in their sera (10–20-fold greater amounts) and the induced IgG freely crossed the placenta to infants (maternal-cord ratios 0.8 and 1.4, respectively) and III CPS-specific IgG in the sera from one- and two-month-old infants whose mothers received the vaccine. This data supports European and South African studies that suggest that maternal immunization with a multivalent GBS conjugate vaccine early in the third trimester would elicit sufficient amounts of CPS-specific IgG to prevent peripartum maternal, as well as early- and late-onset GBS infant, disease [14].

The investigational vaccine described above covers three serotypes that are estimated to cause ≥75% of early infant GBS disease [15]. This is encouraging for a continued search for an ideal vaccine against GBS that would cover all ten serotypes. Additionally, Madhi and colleagues have observed no apparent effect on serotype-specific maternal vaginal colonization at delivery [16]. However, it has been established that serum CPS IgG values of ≥1 μg/mL for serotype V and ≥3 μg/mL for serotypes Ia and III were significantly associated with protection against rectovaginal acquisition of the homotypic serotype. A GBS vaccine that induces sufficient capsular serotype-specific antibody in pregnant women could protect against rectovaginal colonization during the latter half of pregnancy [17]. Thus, it is important to investigate the effect of the vaccine on vaginal maternal colonization in future studies.

## Conclusions

Group B streptococcus (GBS) infection in the neonate is a global issue. It can have devastating outcomes such as meningitis, sepsis, and pneumonia. Reliable prophylaxis is necessary to prevent such complications. The current use of intrapartum antibiotics is an efficacious method of reducing vaginal colony burden in GBS-positive pregnant women, thereby, decreasing transmission to the neonate. The benefits of this intervention, however, are restricted to those who can receive it for a duration of four hours. This time restraint often leaves women in preterm and precipitous labor unprotected. Increasing antibiotic resistance to non-beta lactam alternative such as clindamycin and erythromycin is a growing concern with increasing penicillin allergies. Intrapartum antibiotics are also limited in their use to prevent early-onset GBS infection, with no effect on late-onset GBS infection. The development of a vaccine to reduce both early-onset and late-onset GBS provides a supplement and alternative to intrapartum antibiotics, especially to those unable to complete the four-hour duration of antibiotics. An investigational CRM197 trivalent vaccine has shown a good immunogenicity profile against the three most prevalent serotypes and has a great safety profile free of any serious systemic adverse effects. Future studies should focus on investigating the effect of the vaccine on maternal rectovaginal acquisition and subsequent vaginal colonization, as well as the expansion of vaccine coverage to all 10 serotypes identified worldwide in order to facilitate its use in resource-poor areas of the world with inadequate antibiotic availability.
